# Atraumatic Shoulder-Hand Syndrome Presenting as Complex Regional Pain Syndrome Type 1 in an 81-Year-Old Patient With Diabetes Mellitus

**DOI:** 10.7759/cureus.117644

**Published:** 2026-10-08

**Authors:** Francisco M Torres

**Affiliations:** 1 Physical Medicine and Rehabilitation, Morton Plant Hospital, Clearwater, USA

**Keywords:** atraumatic crisp, budapest criteria, complex regional pain, complex regional pain syndrome type 1, crps 1, hand and wrist pain, pain medicine, shoulder-hand syndrome, type 2 diabetes

## Abstract

Complex regional pain syndrome type 1 (CRPS-1), formerly called reflex sympathetic dystrophy, is defined by the absence of a confirmed nerve lesion and accounts for many CRPS cases. "Shoulder-hand syndrome" describes a related but distinct clinical pattern of ipsilateral shoulder and hand involvement that can occur in either CRPS-1 or CRPS-2, the latter defined by a confirmed nerve lesion. CRPS-1 most commonly follows a noxious inciting event, such as fracture, sprain, surgery, or a period of immobilization, that does not involve direct nerve damage. Fully atraumatic presentations are uncommon and represent a diagnostic challenge, particularly in elderly patients with diabetes mellitus, whose overlapping musculoskeletal and neuropathic comorbidities can obscure the underlying pathophysiology. We report a case of an 81-year-old man with a history of long-standing diabetes mellitus who presented to our clinic two months after the onset of his symptoms with progressive pain, swelling, stiffness, and vasomotor and trophic changes of the right shoulder and hand, in the absence of any known inciting event. Examination revealed allodynia, hyperalgesia, decreased skin temperature, erythema, edema, dryness, hair loss, and severe restriction in motion. Plain radiographs and MRI of the hand and shoulder were unremarkable. The inflammatory markers available at the time, including erythrocyte sedimentation rate and C-reactive protein, were normal. The last HbA1c was moderately elevated. Adhesive capsulitis and diabetic cheiroarthropathy were considered given the patient's diabetic status but were excluded on clinical and imaging grounds. The patient fulfilled the Budapest clinical criteria for CRPS-1, with symptoms and signs including the sensory, vasomotor, sudomotor/edema, and motor/trophic domains. After several weeks of physical therapy to no avail, he responded favorably to a high dose of gabapentin. This case underscores the importance of maintaining a high index of suspicion for atraumatic CRPS-1 in older diabetic patients. It illustrates the diagnostic workup required to differentiate it from more common diabetes-associated shoulder-hand pathology.

## Introduction

Complex regional pain syndrome (CRPS) is a chronic pain condition characterized by disproportionate pain and sensory, vasomotor, sudomotor, motor, and trophic disturbances [1]. CRPS is divided into two subtypes based on the presence or absence of a confirmed nerve lesion. CRPS-1, formerly termed reflex sympathetic dystrophy, lacks an identifiable nerve injury and accounts for approximately 90% of the cases. It usually follows a noxious inciting event, such as a fracture, sprain, surgery, or immobilization, without direct nerve damage. CRPS-2, formerly termed causalgia, is specifically defined by a confirmed peripheral nerve lesion [2]. The historical roots of this distinction trace back to Mitchell et al., who first described causalgia in soldiers with direct nerve injuries during the American Civil War [3].

Steinbrocker introduced the term "shoulder-hand syndrome" in 1947 to describe painful ipsilateral shoulder and hand dysfunction with swelling and, in some cases, permanent trophic changes [4]. Steinbrocker attributed the syndrome most often to coexisting medical conditions rather than to direct nerve trauma, and noted reflex sympathetic dystrophy as an alternative designation [4,5]. Shoulder-hand syndrome is therefore not synonymous with CRPS-1 specifically. Rather, it describes a clinical pattern of ipsilateral shoulder and hand involvement that may arise within either CRPS subtype, depending on whether a nerve lesion is present.

The precise mechanism of CRPS remains incompletely understood. Proposed contributors include an exaggerated local inflammatory response with release of pro-inflammatory neuropeptides; peripheral and central sensitization of nociceptive pathways; dysregulation of the sympathetic nervous system leading to abnormal vasomotor tone; and maladaptive cortical reorganization affecting the representation of the affected limb [6]. This multifactorial pathophysiology explains why CRPS produces the wide, seemingly disconnected constellation of sensory, vasomotor, sudomotor, and motor/trophic findings that characterize the syndrome.

Within CRPS-1, presentations that arise without any identifiable inciting event, whether a non-neural injury, surgery, or a period of immobilization, are comparatively uncommon and easy to overlook or misattribute [7]. This diagnostic difficulty is compounded in elderly patients with long-standing diabetes mellitus, a population prone to several conditions that can mimic the shoulder-hand distribution of CRPS-1, most notably adhesive capsulitis of the shoulder and diabetic cheiroarthropathy (limited joint mobility syndrome) of the hand [8-10]. Diabetic cheiroarthropathy alone has been reported in 8-50% of patients with long-standing diabetes and is diagnosed principally based on the prayer and tabletop signs [8,9]. Because both conditions can coexist with, precede, or coincidentally accompany CRPS-1 in the same patient, clinicians must actively distinguish overlapping diabetic musculoskeletal disease from a distinct atraumatic pain amplification syndrome.

The current reference standard for CRPS-1 diagnosis is the Budapest clinical criteria, developed by Harden et al. and validated as substantially more specific than the earlier International Association for the Study of Pain (IASP) criteria while preserving high sensitivity [11,12]. These criteria require continuing pain that is disproportionate to any inciting event, reported symptoms in at least three of four categories, documented signs in at least two of these four categories on examination, and no other diagnosis that better explains the presentation [11,12]. We present a case in which methodical application of these criteria, alongside targeted exclusion of the two principal diabetic mimickers, established a diagnosis of atraumatic CRPS-1 in an 81-year-old man. This presentation and age group remain sparsely represented in the literature.

## Case presentation

An 81-year-old man with a long-standing history of type 2 diabetes mellitus and no other significant past medical history presented for initial evaluation two months after the onset of progressive pain, swelling, and functional limitation of the ipsilateral right shoulder and hand. He denied any preceding trauma, surgery, fracture, immobilization, cardiac event, cerebrovascular event, or other identifiable inciting event.

The patient's diabetes, present for more than 10 years, was treated adequately with 1000 mg metformin and 10 mg glipizide daily. Two months of treatment for right-sided shoulder, arm, and hand pain with oral prednisone 5 mg once per day and local steroid injections into trigger points did not improve symptoms, leading to the patient's decision to seek treatment from a pain management specialist.

On examination, the affected limb showed allodynia and hyperalgesia, decreased skin temperature of more than 1°C compared with the contralateral side, erythema, edema, skin dryness, and hair loss, along with severe restriction of shoulder and hand range of motion. Skin hypertrophy was noted, with no changes to the fingernails (Figures 1, 2).

**Figure 1:**
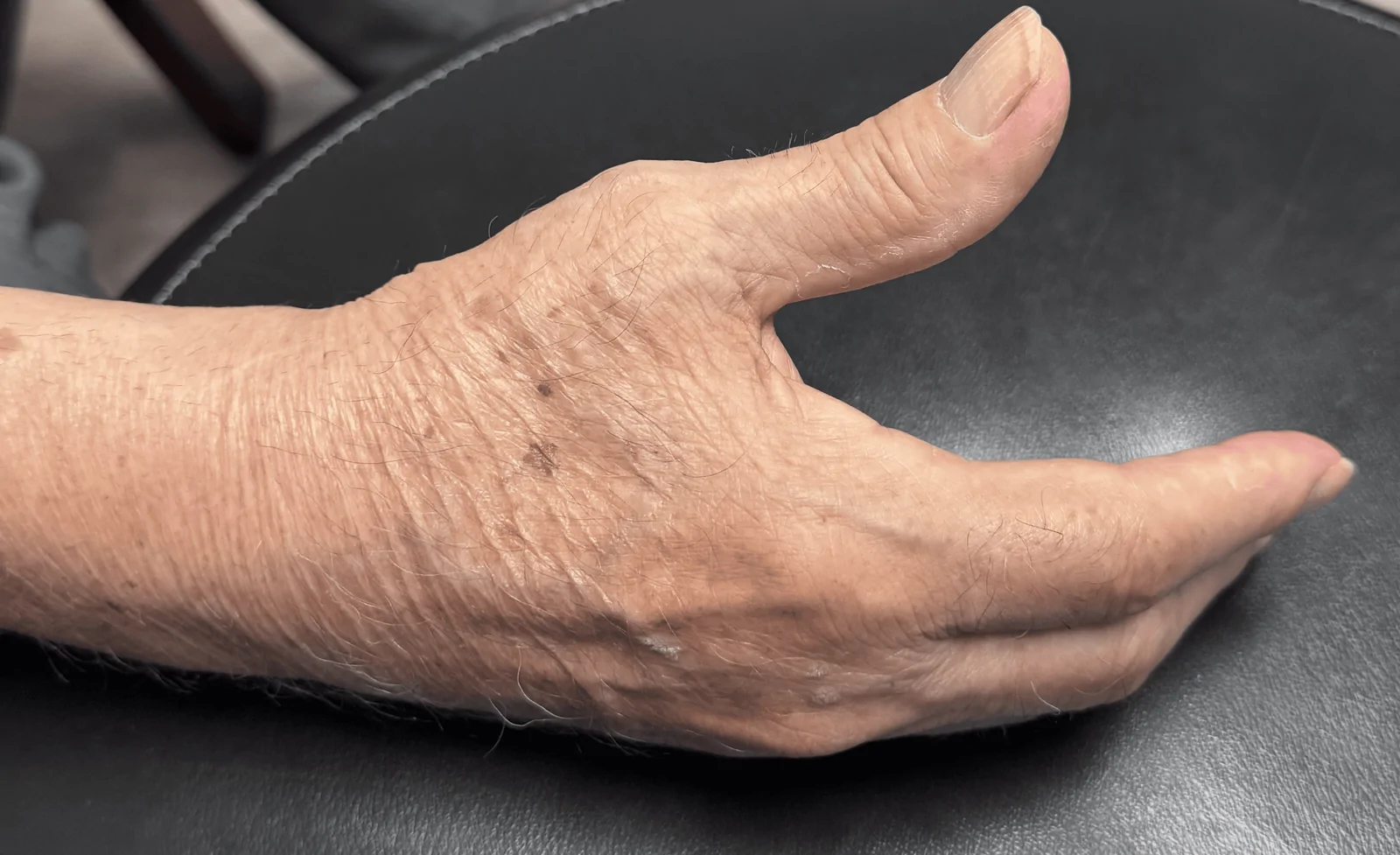
Dorsal/lateral view of the affected hand demonstrating fixed thumb flexion, severe restriction of digital extension, and trophic skin changes (dryness, hair loss).

**Figure 2:**
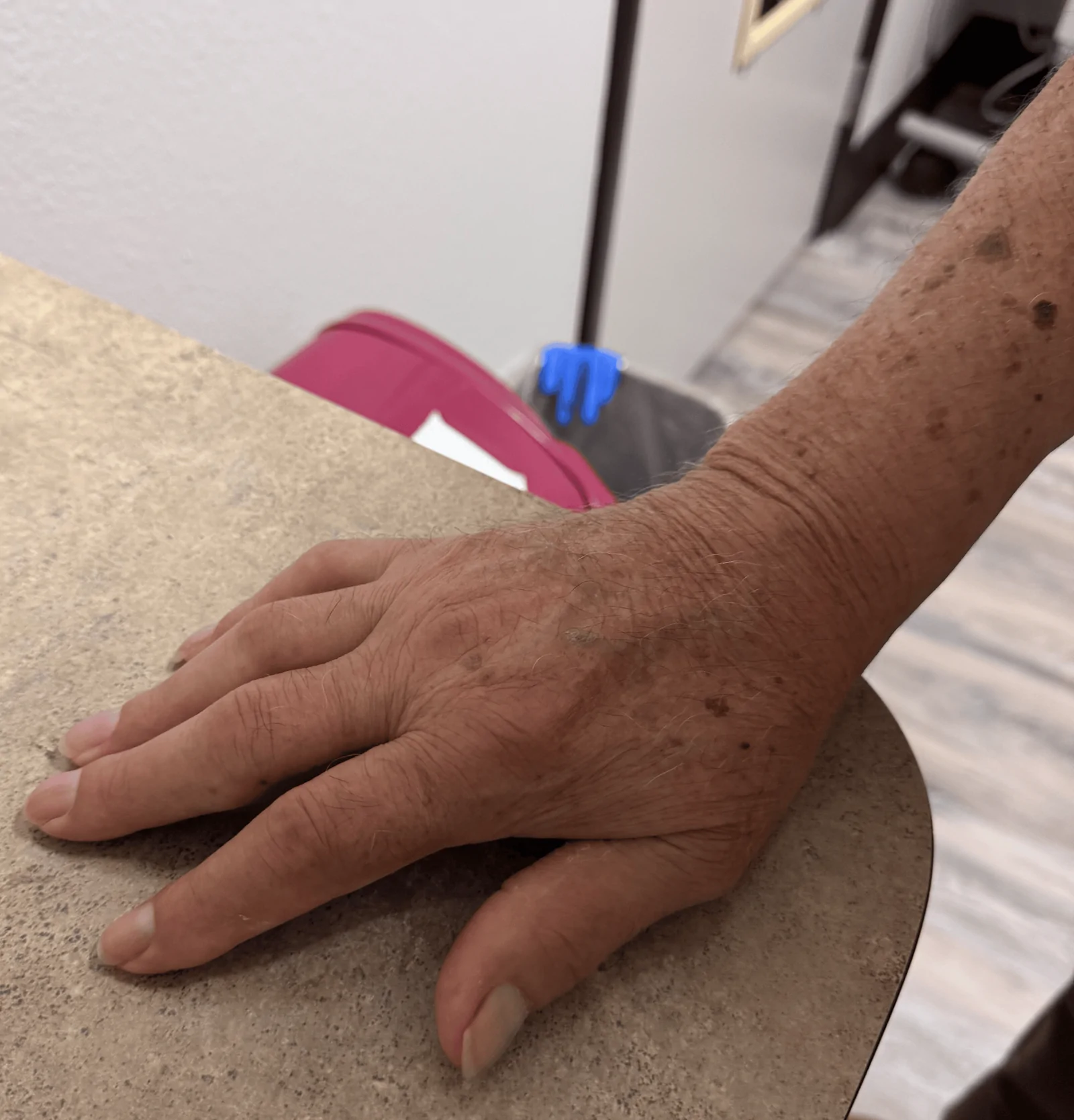
Dorsal view of the unaffected, contralateral (left) hand, shown for comparison, demonstrating normal skin color, full digital extension, and no evidence of edema or trophic change.

Range of movement findings were as follows: shoulder extension 120°, abduction 61°, internal rotation 88°, external rotation limited. Muscle strength was 2/5 during flexion and abduction; 3/5 during extension and external rotation; and 4/5 during flexion, extension, and internal rotation. The patient could not complete the grip strength test due to limited range of motion. Table 1 summarizes pain, range of motion, motor strength, and grip findings. The unaffected left hand, shown for comparison, demonstrated full digital extension without edema, erythema, or trophic change (Figure 3).

**Table 1:** Pain, range-of-motion, and motor strength findings at presentation. DASH: disabilities of the arm, shoulder, and hand; MRC: Medical Research Council; ROM: range of motion

Assessment domains	Parameter	Finding
Pain (modified DASH)	At presentation	6/10 (burning quality)
	At worst	7/10
	At best	3/10
	At follow-up (~5 months later, gabapentin 300 mg BID)	3/10
Right shoulder range of motion	Extension	120°
	Abduction	61°
	Internal rotation	88°
	External rotation	Severely restricted
Motor strength (MRC grade)	Right shoulder flexion and abduction	2/5
	Right shoulder extension and external rotation	3/5
	Right shoulder internal rotation	4/5
	Right elbow flexion and extension	4/5
Grip strength	Right hand	Not obtainable (limited by ROM)

**Figure 3:**
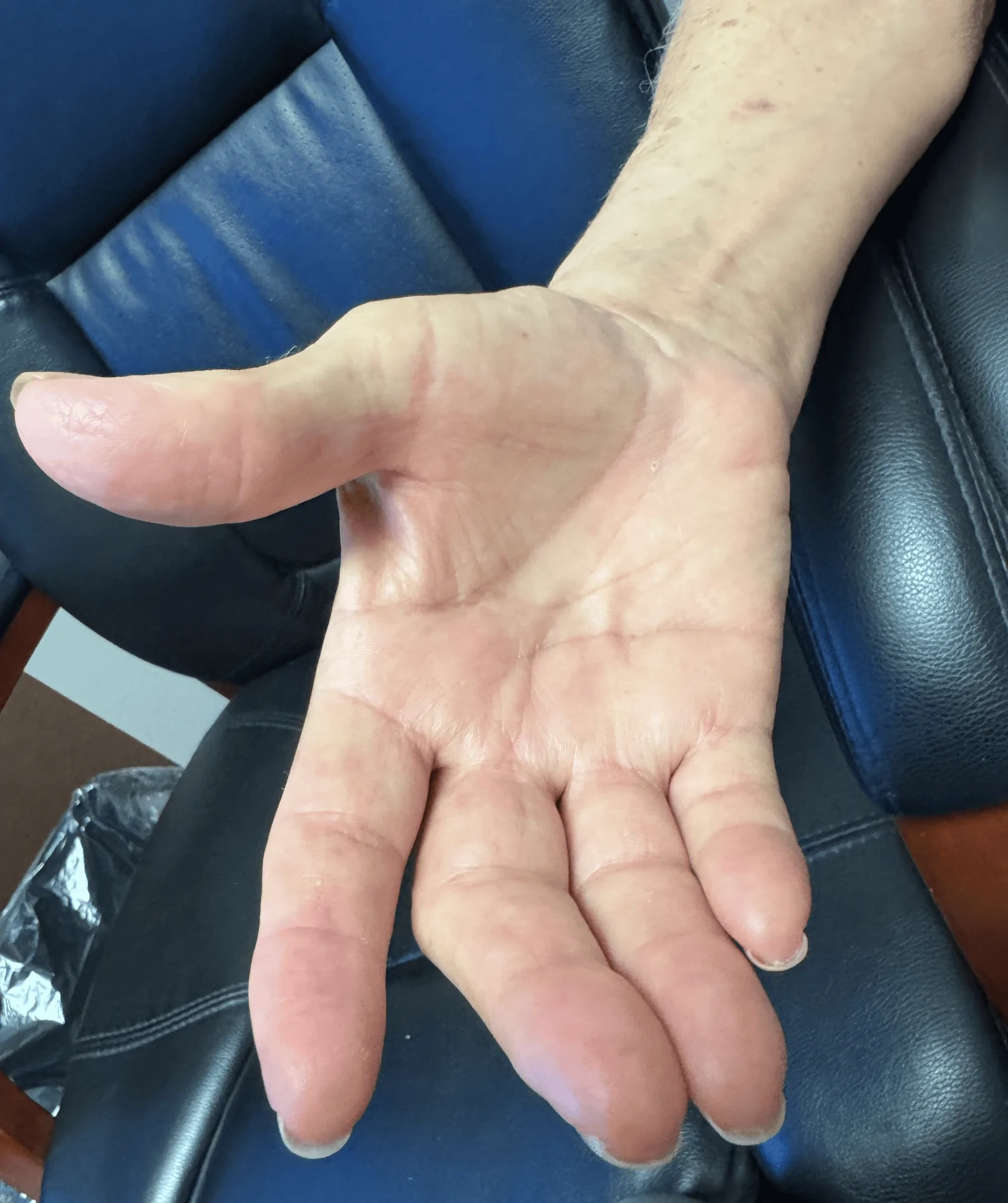
Palmar view of the affected hand demonstrating diffuse erythema, edema, and trophic changes involving the fingers and thenar eminence.

Given the patient's diabetic status, adhesive capsulitis and diabetic cheiroarthropathy were both considered as competing or contributing diagnoses. Adhesive capsulitis was excluded based on MRI findings, including the absence of capsular thickening/synovitis of the axillary recess and rotator interval, and a pattern of motion loss inconsistent with isolated capsular restriction. Diabetic cheiroarthropathy was excluded based on a negative prayer sign and tabletop sign on the left side, and the absence of the classic waxy, thickened dorsal skin change, without other CRPS features.

Plain radiographs of the hand and shoulder MRI were unremarkable, with no evidence of rotator cuff pathology, capsular thickening, or bony abnormality. Laboratory evaluation showed a mildly elevated HbA1c, indicating adequate glycemic control, and normal inflammatory markers (erythrocyte sedimentation rate and C-reactive protein), arguing against an active inflammatory or autoimmune process.

Electromyography (EMG) and nerve conduction studies (NCS), performed about seven months after symptom onset, showed moderate bilateral carpal tunnel and ulnar cubital tunnel nerve compression, along with moderate axonal peripheral neuropathy. None were sufficient to explain the acute onset of observed right-sided symptoms seven months ago. Triple-phase bone scintigraphy was ordered, but the patient's insurance carrier denied it, and it could not be obtained.

The patient's presentation was assessed against the Budapest clinical diagnostic criteria for CRPS-1. He reported symptoms in the sensory (allodynia, hyperalgesia), vasomotor (temperature asymmetry, erythema), sudomotor/edema (edema), and motor/trophic (decreased range of motion, skin/hair trophic change) domains, satisfying the requirement of at least one symptom in three of the four categories. On examination, signs were confirmed in each of the four categories, satisfying the requirement of at least one sign in two of the four categories on examination. No other diagnosis better accounted for the full constellation of findings. The patient therefore fulfilled the Budapest criteria for a clinical diagnosis of CRPS-1.

He was started on gabapentin 300 mg twice daily, titrated to a high dose, with a favorable clinical response in pain control; his average pain score improved from 6/10 at presentation to 3/10 at follow-up approximately five months later (approximately seven months after symptom onset). No other medications were trialed. Concurrent physical therapy produced only mild additional functional improvement.

## Discussion

This case illustrates the following two intersecting diagnostic challenges: the rarity of atraumatic CRPS-1 and the confounding effect of coexisting diabetes in an elderly patient. Within CRPS-1, presentations without an identifiable inciting event are comparatively uncommon, and this absence of a clear precipitant often delays diagnosis, as clinicians reasonably first search for a more common, mechanically explicable cause of shoulder-hand pain [2,7]. In a diabetic patient, that search naturally converges on the following two entities with substantial clinical overlap with CRPS-1: adhesive capsulitis, which produces painful restriction of shoulder motion and is more prevalent in diabetic populations, and diabetic cheiroarthropathy, which produces painless to mildly painful hand stiffness from advanced glycation end-product-mediated collagen cross-linking and is diagnosed principally through the prayer and tabletop signs [8,9]. Neither condition, however, is expected to produce the combination of allodynia, vasomotor asymmetry, edema, and trophic skin/hair change that defines CRPS-1; their exclusion in this patient rested on the absence of imaging findings specific to capsulitis and the absence of the characteristic limited joint mobility signs of cheiroarthropathy, alongside normal inflammatory markers arguing against an active inflammatory joint process.

The Budapest criteria remain the most specific validated tool for this differentiation, requiring symptoms across at least three of four domains (sensory, vasomotor, sudomotor/edema, motor/trophic) and signs across at least two of four domains on examination (Table 2) [11,12]. This patient's presentation mapped cleanly onto all four domains both by history and examination, supporting a robust clinical diagnosis independent of confirmatory imaging. Triple-phase bone scintigraphy, while classically associated with CRPS-1, has been shown in more recent Bayesian meta-analysis to have limited sensitivity when the Budapest criteria are used as the reference standard, and a negative scan does not exclude the diagnosis [13]. In this case, we could not obtain the scan because the patient's insurance carrier denied it, a real-world barrier that clinicians managing atraumatic CRPS-1 in older patients may encounter, particularly when imaging is requested to support rather than establish a Budapest criteria-based diagnosis. We acknowledge that this precludes contribution of nuclear medicine reference data for this specific atraumatic, diabetes-associated presentation, which could otherwise have added diagnostic and comparative value for future case series.

**Table 2:** Budapest clinical diagnostic criteria for CRPS. CRPS: complex regional pain syndrome

Criteria	Requirement
Continuing pain	Disproportionate to any inciting event
Reported symptoms	At least 1 symptom in 3 of 4 categories: sensory (hyperalgesia/allodynia); vasomotor (temperature asymmetry/skin color change); sudomotor/edema (edema and/or sweating changes/asymmetry); motor/trophic (decreased range of motion, motor dysfunction, and/or trophic changes to skin, hair, or nails)
Examination signs	At least 1 sign in 2 of 4 categories (same four categories as above, confirmed on examination)
Exclusion	No other diagnosis better explains the signs and symptoms

Electromyography (EMG) and nerve conduction studies (NCS), performed approximately seven months after symptom onset, identified bilateral median nerve mononeuropathies at the wrist, a right ulnar mononeuropathy at the elbow, and a moderate axonal and demyelinating peripheral neuropathy, findings that are themselves common in patients with long-standing diabetes and do not, in our assessment, better explain this patient's presentation than CRPS-1. The Budapest criteria require that no other diagnosis better accounts for the patient's signs and symptoms. Focal entrapment neuropathies and distal symmetric polyneuropathy characteristically produce sensory loss, paresthesia, or weakness confined to specific nerve distributions rather than the diffuse allodynia, hyperalgesia, vasomotor asymmetry, edema, and trophic skin and hair changes spanning the entire shoulder-hand region observed in this patient. We interpret these EMG findings as coexisting, diabetes-related comorbidities that likely contributed to, but do not fully account for, the sensory component of the presentation, underscoring the importance of evaluating the complete Budapest symptom and sign profile rather than any single finding in isolation when peripheral neuropathy and CRPS-1 coexist in a diabetic patient.

Age is a further point of interest as follows: atraumatic CRPS-1 in a patient in his ninth decade is infrequently reported, and most published shoulder-hand syndrome and CRPS-1 case literature skews toward younger or post-traumatic/post-surgical cohorts. This case adds to a limited body of literature suggesting that atraumatic CRPS-1 should remain on the differential for shoulder-hand pain in older patients even in the absence of a mechanical trigger, and that diabetes itself, through mechanisms including microvascular and small-fiber nerve involvement, may plausibly lower the threshold for its atraumatic development. This relationship remains speculative, and it's not established in a single case.

Finally, the patient's favorable response to high-dose gabapentin is consistent with the neuropathic pain component of CRPS-1. It supports gabapentinoids as a reasonable first-line pharmacologic option in this population, particularly where the extent of joint restriction limits the marginal benefit obtainable from physical therapy alone.

This case is distinct from other recently published atraumatic CRPS reports. Abuzied et al. described a 39-year-old man with a seven-year history of atraumatic right shoulder-hand pain and an otherwise unremarkable medical history, in whom serial cervical spine, shoulder, elbow, and wrist imaging excluded structural pathology [7]. Still, the diagnosis was reached largely by exclusion, without a diabetic comorbidity to confound the clinical picture, and the patient's pain remained refractory to surgical and pharmacologic treatment [7]. By contrast, the present case centers on an elderly patient with long-standing diabetes mellitus, in whom two specific, diabetes-associated conditions, adhesive capsulitis and diabetic cheiroarthropathy, had to be actively differentiated from CRPS-1 using targeted clinical signs and imaging; the Budapest criteria were applied systematically rather than reached by exclusion alone, and the patient achieved a favorable treatment response. These differences underscore that traumatic CRPS-1 is not a single, uniform diagnostic problem - the required differential reasoning changes substantially with patient age and comorbidity profile.

Limitations

This single-case study limits generalizability. The patient's insurance carrier denied triple-phase bone scintigraphy, so it could not be obtained. Although not required to satisfy the Budapest criteria, its absence means this report cannot provide nuclear medicine reference data for this specific atraumatic, diabetes-associated presentation, limiting its value for clinicians seeking a complete comparative diagnostic profile. The coexisting entrapment neuropathies and peripheral polyneuropathy identified on EMG/NCS reflect common diabetes-related comorbidities that complicate, though do not preclude, the diagnostic picture, and their presence should prompt caution against attributing all sensory findings in similar patients to CRPS-1 alone.

## Conclusions

Atraumatic CRPS-1 is an uncommon but clinically important consideration in elderly patients with diabetes mellitus who present with shoulder-hand pain, swelling, and functional limitation in the absence of an inciting event. Because adhesive capsulitis and diabetic cheiroarthropathy are both more prevalent in this population and can superficially resemble CRPS-1, a systematic application of the Budapest clinical criteria, supported by targeted exclusion of these mimickers through examination and imaging, is essential to reach an accurate diagnosis. Maintaining a high index of suspicion for atraumatic CRPS-1 in this setting allows for timely initiation of effective neuropathic pain management, as illustrated by this patient's favorable response to gabapentin.
